# Methodological and reporting quality evaluation of Buyang Huanwu decoction for experimental cerebral ischemia–reperfusion injury: a systematic review

**DOI:** 10.1007/s00210-022-02362-9

**Published:** 2023-01-13

**Authors:** Xiangyu Chen, Tong Yang, Yanan Luo, Zhitao Feng, Rui Fang, Jinwen Ge, Zhigang Mei

**Affiliations:** 1 The Key Laboratory of Hunan Province for Integrated Traditional Chinese and Western Medicine On Prevention and Treatment of Cardio-Cerebral Diseases, College of Integrated Traditional Chinese and Western Medicine, Hunan University of Chinese Medicine, Changsha, 410208 Hunan China; 2 Third-Grade Pharmacological Laboratory On Chinese Medicine Approved By State Administration of Traditional Chinese Medicine, Medical College of China Three Gorges University, Yichang, 443002 Hubei China

**Keywords:** Methodological and reporting quality, Buyang Huanwu decoction, Cerebral ischemia–reperfusion injury, SYRCLE, ARRIVE

## Abstract

Buyang Huanwu decoction, a classic traditional Chinese prescription, has been used to prevent and treat stroke for hundreds of years. An increasing number of the laboratory research on Buyang Huanwu decoction used in treating cerebral ischemia–reperfusion injury have been published recently. However, the problem of methodological and reporting quality of some studies is lack of assessment. This study aims to evaluate the methodological and reporting quality of the research on Buyang Huanwu decoction against experimental cerebral ischemia–reperfusion injury. A comprehensive search on six databases was performed. Two researchers independently screened the literature considering the eligibility criteria. Methodological and reporting quality of the included studies were evaluated by the Systematic Review Centre for Laboratory Animal Experimentation (SYRCLE) risk-of-bias tool and Animal Research: Reporting of In Vivo Experiments (ARRIVE) guideline. Forty-five studies met the inclusion criteria. No study achieved a decent overall rating in using the SYRCLE tool (percentage of items with “low risk” ≥ 50%). Of the 22 items on the SYRCLE tool, only 7 items (31.82%) were rated as “low risk” in more than 50% of the included studies. Of the 39 items of ARRIVE guideline, 14 (35.9%) items were rated as “yes” in more than 50% of the included studies. The methodological and reporting quality of Buyang Huanwu decoction for experimental cerebral ischemia–reperfusion injury was substandard, which needed to be further improved. The limitations should be addressed when planning similar studies in the future. Additionally, these findings provided evidence-based guidance for future preclinical studies evaluating the efficacy of Buyang Huanwu decoction in the treatment of cerebral ischemia–reperfusion injury.

## Introduction

Preclinical research, particularly important to biomedical research, forms the foundation on which future studies are built. The exciting and new ideas it provides will eventually turn into clinical studies and new drugs that provide benefit to humankind. However, some preclinical research is poorly predictive of ultimate success in the clinic owing to the low methodology and reporting quality (Hackam and Redelmeier, 2006; Perel et al. 2007). Indeed, a structured approach or full details in the randomization steps, baseline characteristic balance, blinding procedures, sample size calculations, or sufficient reporting of an experiment allow for reproducibility of the findings. However, an increasing number of evidence showed highly inadequate methodological and reporting quality upon animal research in some scientific publications (Kilkenny et al. 2012). Thus, there is a growing need for valid, efficient, and easy scoring scales and systematic assessment to provide rigorous scientific methods and rate the quality of animal studies. At present, a few studies have assessed the quality of experimental methods and reports by evaluating compliance with various assessment tools (Fabian-Jessing et al. 2018). The Systematic Review Centre for Laboratory Animal Experimentation (SYRCLE), based on the Cochrane Collaboration risk-of-bias (RoB) tool, is an assessment instrument to evaluate the risk of bias and the methodological quality of animal studies (Hooijmans et al. 2014). The Animal Research: Reporting In Vivo Experiments (ARRIVE), a comprehensive set of guidelines for animal research, provides format and content of details relating to animals in a typical scientific report (Kilkenny et al. 2012). Thus, adherence to the two comprehensive sets of rules for inclusion of detail that is consistent across all articles has many advantages (McGrath et al. 2010).

Worldwide, stroke is an important cause of disability and mortality. The burden of stroke has increased substantially over the past few decades due to population growth and aging as well as the increased prevalence of modifiable stroke risk factors, especially in developing countries (Katan and Luft, 2018). At present, there are three different types of strokes: Ischemic strokes, hemorrhagic strokes, and transient ischemic attacks, among which ischemic strokes account for about 87% of all strokes (Virani et al. 2020). Ischemic stroke is characterized by the sudden loss of blood circulation to an area of the brain, typically in a vascular territory, resulting in a corresponding loss of neurologic function (Virani et al. 2020). Currently, FDA-approved drug for ischemic stroke is the recombinant tissue plasminogen activator (r-tPA), a fibrinolytic agent, which is effective if applied within 3 h, but no longer than 4.5 h, after symptom onset (Rabinstein, 2017). However, cerebral ischemia–reperfusion injury (CIRI) following the application of r-tPA sometimes will lead to secondary injury to the brain tissue. The short therapeutic time window and CIRI limited the benefit of r-tPA for large clot burden, and hence, research is ongoing to find more effective and safer reperfusion therapy, as well as focusing on refinement of patient selection for acute reperfusion treatment (Fukuta et al. 2017).

According to the theory of traditional Chinese medicine (TCM), the primary pathological process of ischemic stroke is *Qi* deficiency and blood stasis, and *Qi* is an important concept in the theory of TCM, which is a vital energy that can invigorate the body and promote blood circulation and meridian circulation (Li et al. 2014; Tan et al. 2016; Zhao et al. 2012). Buyang Huanwu decoction (BHD), a classic traditional Chinese prescription invented by the Chinese well-known herbalist Wang Qing-ren (AD 1768–1831) for the treatment of ischemic stroke with *Qi* deficiency and blood stasis, exhibits the efficacy of replenishing *Qi* and activating blood circulation according to the theory of TCM. It has been utilized clinically and showed significant preventive and therapeutic effects for ischemic stroke and stroke-induced disability for more than 190 years in China and some other Asian countries (Wei et al. 2013). Recently, increasing clinical evidence about BHD application have showed significant improvement in neural functions and symptoms for ischemic stroke (Han et al. 2018; Hao et al. 2012; Jiang et al. 2020; Li et al. 2014), while the underlying mechanisms remain indistinct. BHD consists of seven kinds of Chinese medicinal materials: Radix Astragali, Radix Paeoniae Rubra, Radix Angelicae Sinensis, Rhizoma Ligustici Chuanxiong, Flos Carthami, Semen Persicae, and Pheretima Aspergillum (Table 1) (Cui et al. 2015). Growing experimental studies reported that BHD was beneficial for cerebral ischemia and CIRI, suggesting that BHD may be a prospective therapy that could decrease infarct volume and ameliorate neurological impairment (Cai et al. 2007; Chen et al. 2020a, b, 2019). There are basic methodology and reporting practices in the laboratory study upon BHD treatment against CIRI that are sub-optimal, and it is likely to be affecting the validity and replicability of research.

**Table 1:** The ingredients of Buyang Huanwu decoction

Latin name	English name	Chinese name	Family	Part used
Radix Astragali	*Astragalus mongholicus*	Huang qi	Leguminosae	Root
Radix Paeoniae Rubra	Red peony root	Chi shao	Ranunculaceae	Root
Radix Angelicae Sinensis	Angelica root	Dang gui	Umbelliferae	Root
Rhizoma Ligustici Chuanxiong	Sichuan lovage rhizome	Chuan xiong	Umbelliferae	Root
Flos Carthami	*Carthamus tinctorius*	Hong hua	Feverfew	Flower
Semen Persicae	Peach seed	Tao ren	Rosaceae	Seed
Pheretima Aspergillum	Earthworm (*Lumbricus*)	Di long	Megascolecidae	Whole animal

Thus, the aim of this study was to assess the methodological and reporting quality of experimental researches concerning BHD treatment for CIRI with the SYRCLE tool and ARRIVE guideline, respectively, to provide valuable insights for future studies and support the development of methodological and reporting guidance.

## Methods

### Search strategies

Studies of BHD in animal models of CIRI were identified from PubMed, Embase, China National Knowledge Infrastructure (CNKI), VIP Database for Chinese Technical Periodicals (VIP), China Biology Medicine Database (CBM), and Wan Fang Data until November 23, 2022. Our search strategy included the following words and phrases: “Buyang Huanwu” OR “Bu yang Huan wu” OR “Bu-yang Huan-wu” AND “cerebral ischemia–reperfusion.”

### Inclusion criteria and data extraction

An eligible study had to meet all of the following criteria: (1) The retrieval date was from January 1, 2015 to November 23, 2022; (2) only journal articles were selected; (3) experimental models of CIRI were induced in rats; (4) Buyang Huanwu decoction was used as treatment; and (5) articles published in Chinese or English languages were included. Studies were excluded if they were in vitro studies, clinical articles, review comments, publication without full text, duplicated researches, and language other than English or Chinese. The following information was extracted using a predesigned data extraction form from each eligible study: first author, year of publication, strain, sex, weight, anesthetic, method of establishing the model, and effects of BHD. All the included studies were checked for the consistency separately by two assessors (XY Chen, T Yang), and disagreement would be settled by discussion and a third assessor (ZG Mei).

### Methodological and reporting quality evaluation

Two assessors (XY Chen, T Yang) were trained to assess the methodological and reporting quality by experienced assessors before the assessment started. Each reviewer independently assessed the quality of each study. Methodological quality evaluation was assessed using the SYRCLE tool. According to the SYRCLE tool, each item was assigned one of three responses: “low risk, unclear or high risk.” Reporting quality evaluation was performed using the ARRIVE guideline. According to the satisfaction degree of item reporting requirements, it can be divided into “yes,” “partial yes,” and “no.” In the case of a discrepancy, each reviewer provided reasoning for the judgment and disagreements were solved by discussion. If necessary, the third assessor (ZG Mei) was involved in judgment.

### Data analysis

Microsoft Excel 2016 was used for the descriptive statistical analysis, and summary statistics were given percentages. Kappa test was performed by utilizing SPSS 25.0 (IBM Corp., Armonk, NY, USA). The kappa index was used to measure the inter-rater reliability between the two assessors for the SYRCLE tool and ARRIVE guideline. A kappa index less than 0.4 suggested poor agreements, 0.4 to 0.75 suggested fair agreements, and over 0.75 suggested excellent agreements.

## Results

### Study selection

**Fig. 1:**
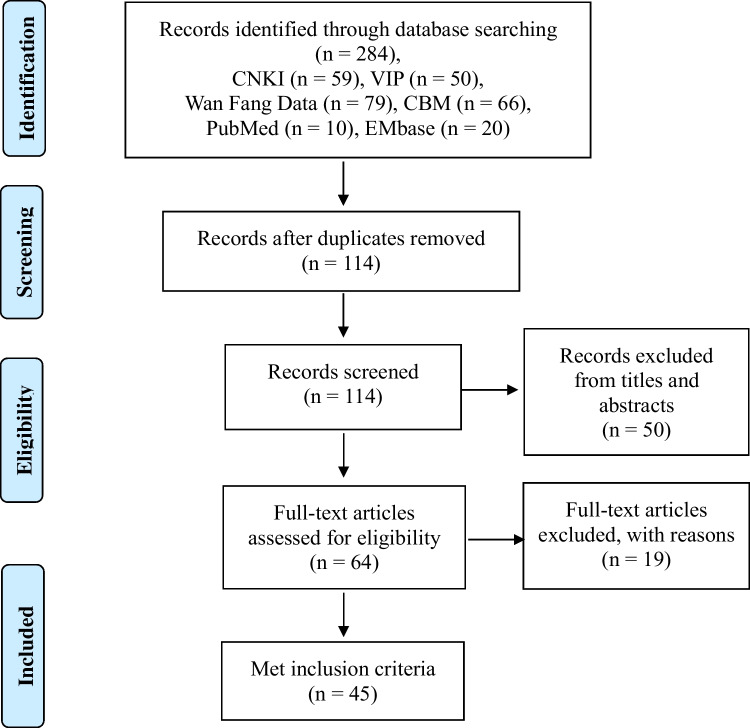
Flow chart of literature search

The search strategies yielded 284 records in total. After duplicating retrieval by the NoteExpress database, 114 studies were remained. Among these, 50 records were excluded due to failing to meet the inclusion criteria after screening the titles and abstracts. The full texts of the remaining 64 records were examined for further assessment. And, 19 studies were discarded because of duplicated publication and improper indices. Finally, we included 45 studies in this overview. A flow chart describing the systematic search and study selection process is shown in Fig. 1.

### Basic characteristics of included studies

Among the included studies, about 80% of studies were published in Chinese-language journals, and the remaining 9 studies were in English language. In terms of strain and sex of rats, 35 studies used male of Sprague Dawley, 1 study utilized male of Wistar, and 4 studies used female and male of Sprague Dawley. Five studies used Sprague Dawley without indicating sex of rats. The weight of rats varied from 150 to 320 g in each study. There were 27 studies anesthetized rats with chloral hydrate, 8 studies with pentobarbital sodium, 4 studies with isoflurane, 1 study with 3% amobarbital sodium, 1 study with Zoletil 50 and xylazine, and the remaining 4 studies not listed in the article. Different methods of modeling were performed, including middle cerebral artery occlusion, 4-vessel occlusion, carotid artery drainage, and bilateral carotid artery occlusion. Different outcome indexes were observed, and the most frequently used indicator was neurological severity score (in 19 studies); others included cerebral infarct size, cerebral edema volume, Bcl-2, Bax, VEGF, AKT, p-AKT, IL-6, SOD, and so on. The main characteristics of including studies are displayed in Table 2.

**Table 2:** The characteristics of the included studies

Author (year)	Strain/sex	Weight (g)	Anesthetic	Modeling methods	Ischemia	Reperfusion	Effects of BHD
Yang et al. (2020)	SD/M	250–300	10% chloral hydrate	Middle cerebral artery occlusion	2 h	7 days	SYN ↑; GAP-43 ↑
Liang et al. (2019)	SD	280–350	–	4-vessel occlusion	15 min	2 h; 12 h; 1 day; 2 days; 3 days	Cerebral edema volume ↓; MDA ↓; SOD ↑; L-type Ca^2+^ ↓
Zhang et al. (2019b)	SD/M	180–220	10% chloral hydrate	Middle cerebral artery occlusion	2 h	14 days	Neurological function score ↓; cerebral infarct size ↓; TUNEL ↓; MDA ↓; SOD ↑; GPx ↑; caspase-3 ↓, caspase-8 ↓, and caspase-9 ↓; Bax ↓; Bcl-2 ↑
Zhou et al. (2019)	SD/M	250–300	10% chloral hydrate	Middle cerebral artery occlusion	2 h	7 days	BWT ↓; SAB test ↑; MVD ↑
Ma and Xie (2018)	SD/M	260–320	–	Bilateral carotid artery occlusion	1 h	1 h	p-AKT-positive cell ↑; p-AKT ↑
Li (2018)	SD/M	151–250	10% chloral hydrate	Middle cerebral artery occlusion	2 h	–	TXB2 ↓; 6-keto-PGF1α ↑; PT ↑; APTT ↑; TT ↑; FIB ↓
Guo et al. (2017)	SD/M	220–250	10% chloral hydrate	Middle cerebral artery occlusion	2 h	1 day; 7 days	IL-1β ↓ (1 day); IL-10 ↑ (1 day); Cx43 ↓ (1 day); Cx43 ↑ (7 days); bFGF ↑ (7 days)
Wei et al. (2017)	SD	300 ± 35	3% amobarbital sodium	Carotid artery drainage	15 min × 3 times	7 days	Neurological function score ↓; Drp1 ↓; Fis1 ↓; cyt-c ↓; Ca^2+^ ↓
Xu et al. (2017)	SD/M	250–320	10% chloral hydrate	Middle cerebral artery occlusion	2 h	6 h	Neurological function score ↓; cerebral infarct size ↓; p-Src ↓; p-Akt ↓; p-p38 MAPK ↓
Ding et al. (2017)	SD/M, F	250–320	10% chloral hydrate	Bilateral carotid artery occlusion	0.5 h	1 day	ECG ↑; cerebral edema volume ↓; LDH ↓; MDA ↓; GSH-Px ↑; TUNEL ↓
Wang et al. (2016)	SD/M	180 ± 20	–	Middle cerebral artery occlusion	–	–	Neurological function score ↓; FLUX ↑; 6-keto-PGF1α ↑ (H)
Huang et al. (2016)	SD/M	180 ± 20	10% chloral hydrate	Middle cerebral artery occlusion	–	14 days	TNF-α ↓; IL-6 ↓; IL-8 ↓; NF-κB ↓; ROS ↓
Shi and Zhou (2016)	W/M	220 ± 10	1% pentobarbital sodium	Bilateral carotid artery occlusion	10 min × 3 times	8 days	Stroke index ↓; 5-HT ↑
Zheng et al. (2016)	SD/M	280–350	10% chloral hydrate	Middle cerebral artery occlusion	2 h	6 h	Neurological function score ↓; cerebral infarct size ↓; CD62P ↓; P-Akt ↓; P-PDK1 ↓
Lai et al. (2016)	SD/M	250–300	Chloral hydrate	Middle cerebral artery occlusion	–	–	Cerebral infarct size ↓; GSK3β mRNA ↓; CD63 ↓
Tu et al. (2015)	SD/M	250 ± 20	10% chloral hydrate	Middle cerebral artery occlusion	2 h	10 days; 19 days; 28 days	Hes1 ↑ (early); Hes5 ↑ (early); Hes1 ↓ (late); Hes5 ↓ (late)
Wang et al. (2015)	SD/M, F	250–300	10% chloral hydrate	Bilateral carotid artery occlusion	1 h	1 h	WBV ↓; PAF ↓; CD62p ↓; CD63 ↓
Zhu et al. (2015b)	SD/M	250 ± 20	10% chloral hydrate	Middle cerebral artery occlusion	2 h	7 days	SOD ↑; GST ↑; GSH-PX ↑; MDA ↓
Cai et al. (2015)	SD	250–300	10% chloral hydrate	Bilateral carotid artery occlusion	1 h	1 h	AKT ↑; p-AKT ↑
Qiu et al. (2015)	SD	220–250	10% chloral hydrate	Middle cerebral artery occlusion	2 h	1 day; 3 days; 7 days	Cx43 ↓ (1 day); Cx43 ↑ (3 days); Cx43 ↑ (7 days); GFAP ↓
Zhu et al. (2015c)	SD/M	250–300	10% chloral hydrate	Middle cerebral artery occlusion	1 h	12 h	Neurological function score ↓; CD62P ↓; PTEN mRNA ↓
Guan et al. (2015)	SD/M, F	180–220	Pentobarbital sodium	4-vessel occlusion	15 min	2 h; 24 h	NR2B mRNA ↓
Zhu et al. (2015a)	SD/M	250–300	10% chloral hydrate	Middle cerebral artery occlusion	1 h	6 h	Neurological function score ↓; cerebral infarct size ↓; p-Akt ↑
Ye et al. (2022)	SD/M	260–270	1% pentobarbital sodium	Middle cerebral artery occlusion	2 h	3 days	mNSS score ↓; cerebral infarction volume ↓
Cheng et al. (2022)	SD	230.3 ± 27.2	2.5% pentobarbital sodium	Middle cerebral artery occlusion	1 h	7 days	Henderson behavioral score ↓; cerebral infarct size ↓; GRP78 mRNA ↓; PERK mRNA ↓; elF2α mRNA ↓; ATG12 mRNA ↓
Ma et al. (2022)	SD/M	250–280	1% pentobarbital sodium	Middle cerebral artery occlusion	2 h	3 days	Neurological function score ↓; cerebral infarct size ↓; LC3-II/LC3-I ↓; p-mTOR/mTOR ↓; p-AMPK/AMPK ↑; p-ULK1/ULK1 ↑
Xin et al. (2022)	SD/M	250–280	1% pentobarbital sodium	Middle cerebral artery occlusion	2 h	3 days	Neurological function score ↓; cerebral infarct size ↓; SIRT1 ↑; Ac NF-κB p65/NF-κB p65 ↓; IL-6 ↓
Zhang et al. (2021)	SD/M	300–320	2% isoflurane	Middle cerebral artery occlusion	1 h	9 days; 15 days; 30 days	BrdU^+^/disheveled^+^ ↑; BrdU + /β-catenin + cells ↑
Wan et al. (2021)	SD/M	280 ± 10	10% chloral hydrate	Middle cerebral artery occlusion	1 h	1 day	Neurological function score ↓; cerebral infarct size ↓; IκBα ↑; TLR4 ↓; NF-κB p65 ↓
Liu et al. (2021)	SD/M	250–300	10% chloral hydrate	Middle cerebral artery occlusion	2 h	1 day	Neurological behavior ↑; cerebral infarct size ↓; FPR2 ↑; RvD1 ↑
Wu et al. (2021)	SD/M	250–300	–	Middle cerebral artery occlusion	2 h	1 day	Bcl-2 ↑; caspase-3 ↓; Bax ↓; NO ↓; MDA ↓; GSH ↑; SOD ↑; NOX2 ↓
Ma et al. (2021)	SD/M	200 ± 20	1% pentobarbital sodium	Middle cerebral artery occlusion	2 h	7 days	Neurological function score ↓; cerebral infarct size ↓; cerebral edema volume ↓; Wnt3a ↑; Wnt3a mRNA ↑; β-catenin ↑; β-catenin mRNA ↑; cyclin D1 ↑; cyclin D1 mRNA ↑; Bcl-2/Bax ↑; Bcl-2/Bax mRNA ↑
Zhuge et al. (2020)	SD/M	250–300	10% chloral hydrate	Middle cerebral artery occlusion	1.5 h	14 days	Cerebral infarct size ↓; BrdU^+^/DCX^+^ ↑; BrdU^+^/NeuN^+^ ↑; BrdU^+^/vWF^+^ ↑; miR-199a-5p ↑; VEGF ↑; BDNF ↑
Wang et al. (2020)	SD/M	230–250	10% chloral hydrate	Middle cerebral artery occlusion	2 h	14 days	Cerebral blood flow ↑; cerebral edema volume ↓; VEGF ↑; VEGF mRNA ↑
Gan et al. (2019)	SD/M	280–300	10% chloral hydrate	Middle cerebral artery occlusion	1.5 h	14 days	CD86 ↓; iNOS ↓; TNF-α mRNA ↓; IL-1β mRNA ↓; IL-6 mRNA ↓; CD206 mRNA ↑; Arg-1 mRNA ↑; IL-10 mRNA ↑; TGF-β mRNA ↑
Liu et al. (2018)	SD/M	250–300	10% chloral hydrate	Middle cerebral artery occlusion	2 h	24 h	NO ↓; MDA ↓; SOD ↑; Cx43 ↓
Li et al. (2021)	SD/M	270–280	2.5% isoflurane	Middle cerebral artery occlusion	2 h	5 days	Neurological function score ↓; cerebral infarct size ↓; nestin ↑; BDNF ↑; DCX ↑; Beclin-1 ↑; LC3-II ↑; SIRT1 ↑; p62 ↓
Zhao et al. (2021)	SD/M	250–280	1% pentobarbital sodium	Middle cerebral artery occlusion	2 h	1 day; 3 days; 7 days	Cerebral infarct size ↓; Beclin-1 ↓; LC3-II/LC3-I ↓
Shen et al. (2020)	SD/M	220–250	3% isoflurane	Middle cerebral artery occlusion	1.5 h	–	Neurological function score ↓; MWM test ↑; MVD ↑; CBF ↑
Chen et al. (2020a)	SD/M	250–300	Zoletil and xylazine	Middle cerebral artery occlusion	1.5 h	1 day; 7 days; 14 days	Neurological function score ↓; cerebral infarct size ↓; TNF-α ↓; IL-10 ↓
Chen et al. (2019)	SD/M	190–210	10% chloral hydrate	Middle cerebral artery occlusion	1.5 h	1 day	Neurological function score ↓; cerebral infarct size ↓; cerebral edema volume ↓; HIF-1α ↓; VEGF ↓; β-ENaC ↑; HIF-1α mRNA ↓; VEGF mRNA ↓; β-ENaC mRNA ↑
She et al. (2019)	SD/M	220–250	10% chloral hydrate	Middle cerebral artery occlusion	2 h	1 day	Neurological function score ↓; cerebral infarct size ↓; Nissl ↑; TUNEL ↓; NLRP3 ↓; ASC ↓; pro-caspase-1 ↓; caspase-1 ↓; IL-1β ↓
Dou et al. (2018)	SD/M	200–220	2.5% isoflurane	Middle cerebral artery occlusion	1 h	3 days	Neurological function score ↓; cerebral infarct size ↓; BBB ↓; MMP ↓; ZO-1 ↓; occludin ↓; IFN-γ ↓; CXCL10 ↓; CXCR3 ↓; NF-κB ↓
Zheng et al. (2018)	SD/M	250–280	4% chloral hydrate	Middle cerebral artery occlusion	2 h	1 day; 3 days; 7 days; 14 days	Neurological function score ↓; MVD ↑; SIRT1 ↑; SIRT1 mRNA ↑; VEGF ↑; VEGF mRNA ↑
Zhang et al. (2016)	SD/M, F	280–300	10% chloral hydrate	Middle cerebral artery occlusion	2 h	14 days	Neurological function score ↓; cerebral infarct size ↓; VEGF ↑; ITGαvβ3 ↑

Arrow up (↑) indicates upregulation of expression, and arrow down (↓) indicates downregulation of expression

*SD* Sprague Dawley, *W* Wistar, *M* male, *F* female

### Methodological quality of included studies

**Fig. 2:**
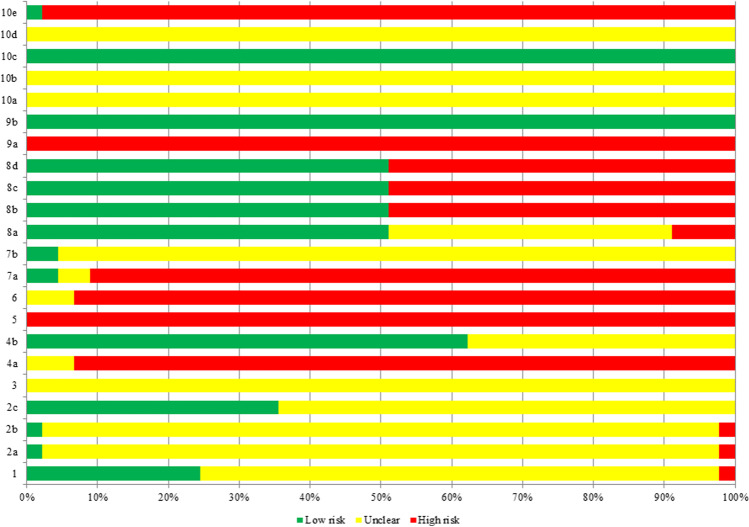
Methodological quality assessment by the SYRCLE tool

General compliance with the SYRCLE tool was incomplete. No study achieved a decent overall rating (percentage of items with “low risk” ≥ 50%) with the SYRCLE tool. Therefore, the items of all the studies were poorly evaluated. Among all the 22 items, merely 2 items about the published report included all expected outcomes, which was rated as low risk bias in all articles. Eleven studies (24%) described a random component in the sequence generation process. One study (2%) kept the distribution of relevant baseline characteristics balanced for the intervention and control groups. Sixteen studies (36%) induced the disease before randomization of the intervention, and twenty-eight studies (62%) mentioned the outcome was not influenced by not randomly housing the animals. When it came to whether the outcome assessor was blinding, two studies (4%) blinded outcome assessor and judged the outcome was not likely to be influenced by lack of blinding. Twenty-three studies held all animals included in the analysis to ensure adequate outcome data. Only one study reported new animals added to the control and experimental groups to replace dropouts from the original population. There were still 8 items that were poorly evaluated, including the allocation of concealment, housing of animals randomly, implementation of blinding between the caregivers and investigators, selection of animals randomly, planning of a protocol and other problems that could result in a high risk of bias. Overall, only 7 items (31.82%) were rated as “low risk” in more than 50% of the included studies of the 22 items on the SYRCLE tool. The inter-rater reliability was excellent between the two assessors (kappa = 0.94). The details can be found in Table 3 and Fig. 2.

**Table 3:** Methodological quality evaluation by the SYRCLE tool

Study	Item																						Of “L” ( %)
	1	2a	2b	2c	3	4a	4b	5	6	7a	7b	8a	8b	8c	8d	9a	9b	10a	10b	10c	10d	10e	
Yang et al. (2020)	U	U	U	L	U	H	L	H	H	H	U	U	H	H	H	H	L	U	U	L	U	H	4 ( 18)
Liang et al. (2019)	L	U	U	L	U	H	U	H	H	H	U	L	L	L	L	H	L	U	U	L	U	H	8 ( 36)
Zhang et al. (2019b)	H	U	U	U	U	H	L	H	H	H	U	L	L	L	L	H	L	U	U	L	U	H	7 ( 32)
Zhou et al. (2019)	U	U	U	L	U	H	U	H	H	H	U	L	L	L	L	H	L	U	U	L	U	H	7 ( 32)
Ma and Xie (2018)	U	U	U	U	U	H	U	H	H	H	U	L	L	L	L	H	L	U	U	L	U	H	6 ( 27)
Li (2018)	L	U	U	U	U	H	L	H	H	H	U	L	L	L	L	H	L	U	U	L	U	H	8 ( 36)
Guo et al. (2017)	U	U	U	L	U	H	U	H	H	H	U	L	L	L	L	H	L	U	U	L	U	H	7 ( 32)
Wei et al. (2017)	U	U	U	U	U	H	L	H	H	H	U	L	L	L	L	H	L	U	U	L	U	H	7 ( 32)
Xu et al. (2017)	L	U	U	U	U	H	U	H	H	H	U	L	L	L	L	H	L	U	U	L	U	H	7 ( 32)
Ding et al. (2017)	U	U	U	U	U	H	L	H	H	H	U	H	H	H	H	H	L	U	U	L	U	H	3 ( 14)
Wang et al. (2016)	U	U	U	U	U	H	U	H	H	H	U	U	H	H	H	H	L	U	U	L	U	H	2 ( 9)
Huang et al. (2016)	L	U	U	U	U	H	U	H	H	H	U	U	H	H	H	H	L	U	U	L	U	H	3 ( 14)
Shi and Zhou (2016)	L	U	U	U	U	H	L	H	H	H	U	L	L	L	L	H	L	U	U	L	U	H	8 ( 36)
Zheng et al. (2016)	U	U	U	U	U	H	U	H	H	H	U	H	H	H	H	H	L	U	U	L	U	H	2 ( 9)
Lai et al. (2016)	L	U	U	U	U	H	U	H	H	H	U	L	L	L	L	H	L	U	U	L	U	H	7 ( 32)
Tu et al. (2015)	U	U	U	U	U	H	L	H	H	H	U	H	H	H	H	H	L	U	U	L	U	L	4 ( 18)
Wang et al. (2015)	L	U	U	U	U	H	U	H	H	H	U	U	H	H	H	H	L	U	U	L	U	H	3 ( 14)
Zhu et al. (2015b)	U	U	U	L	U	H	L	H	H	H	U	L	L	L	L	H	L	U	U	L	U	H	8 ( 36)
Cai et al. (2015)	L	U	U	U	U	H	U	H	H	H	U	U	H	H	H	H	L	U	U	L	U	H	3 ( 14)
Qiu et al. (2015)	U	U	U	L	U	H	U	H	H	H	U	U	H	H	H	H	L	U	U	L	U	H	3 ( 14)
Zhu et al. (2015c)	L	U	U	U	U	H	U	H	H	H	U	U	H	H	H	H	L	U	U	L	U	H	3 ( 14)
Guan et al. (2015)	U	U	U	U	U	H	U	H	H	H	U	U	H	H	H	H	L	U	U	L	U	H	2 ( 9)
Zhu et al. (2015a)	U	U	U	U	U	H	U	H	H	H	U	L	L	L	L	H	L	U	U	L	U	H	6 ( 27)
Ye et al. (2022)	U	U	U	L	U	H	L	H	H	H	U	U	H	H	H	H	L	U	U	L	U	H	4 ( 18)
Cheng et al. (2022)	U	H	H	L	U	H	L	H	H	H	U	L	L	L	L	H	L	U	U	L	U	H	8 ( 36)
Ma et al. (2022)	U	U	U	U	U	H	L	H	H	H	U	L	L	L	L	H	L	U	U	L	U	H	7 ( 32)
Xin et al. (2022)	U	U	U	L	U	H	L	H	H	H	U	U	H	H	H	H	L	U	U	L	U	H	4 ( 18)
Zhang et al. (2021)	L	U	U	U	U	U	L	H	U	U	U	L	L	L	L	H	L	U	U	L	U	H	8 ( 36)
Wan et al. (2021)	U	U	U	L	U	H	L	H	H	H	U	L	L	L	L	H	L	U	U	L	U	H	8 ( 36)
Liu et al. (2021)	U	U	U	L	U	H	L	H	H	H	U	U	H	H	H	H	L	U	U	L	U	H	4 ( 18)
Wu et al. (2021)	U	U	U	L	U	H	L	H	H	H	U	L	L	L	L	H	L	U	U	L	U	H	8 ( 36)
Ma et al. (2021)	U	U	U	L	U	U	L	H	U	U	U	H	H	H	H	H	L	U	U	L	U	H	5 ( 23)
Zhuge et al. (2020)	U	U	U	L	U	H	L	H	H	H	U	U	H	H	H	H	L	U	U	L	U	H	4 ( 18)
Wang et al. (2020)	U	U	U	L	U	H	L	H	H	H	U	L	L	L	L	H	L	U	U	L	U	H	4 ( 18)
Gan et al. (2019)	L	U	U	U	U	H	L	H	H	H	U	U	H	H	H	H	L	U	U	L	U	H	4 ( 18)
Liu et al. (2018)	U	U	U	U	U	H	L	H	H	H	U	U	H	H	H	H	L	U	U	L	U	H	3 ( 14)
Li et al. (2021)	U	U	U	U	U	H	L	H	H	H	U	L	L	L	L	H	L	U	U	L	U	H	7 ( 32)
Zhao et al. (2021)	U	U	U	U	U	U	L	H	U	H	U	U	H	H	H	H	L	U	U	L	U	H	3 ( 14)
Shen et al. (2020)	U	U	U	U	U	H	U	H	H	L	L	U	H	H	H	H	L	U	U	L	U	H	4 ( 18)
Chen et al. (2020a)	U	U	U	U	U	H	L	H	H	H	U	U	H	H	H	H	L	U	U	L	U	H	3 ( 14)
Chen et al. (2019)	U	U	U	U	U	H	L	H	H	H	U	U	H	H	H	H	L	U	U	L	U	H	3 ( 14)
She et al. (2019)	U	L	L	U	U	H	L	H	H	H	U	L	L	L	L	H	L	U	U	L	U	H	9 ( 41)
Dou et al. (2018)	U	U	U	L	U	H	U	H	H	L	L	L	L	L	L	H	L	U	U	L	U	H	9 ( 41)
Zheng et al. (2018)	U	U	U	U	U	H	L	H	H	H	U	L	L	L	L	H	L	U	U	L	U	H	7 ( 32)
Zhang et al. (2016)	U	U	U	U	U	H	L	H	H	H	U	L	L	L	L	H	L	U	U	L	U	H	7 ( 32)

L, low risk; H, high risk; U, unclear

1, sequence generation; 2, baseline characteristics; 3, allocation concealment; 4, random housing;

5, performance blinding; 6, random outcome assessment; 7, detection blinding; 8, incomplete outcome data;

9, selective outcome reporting; 10, other sources of bias

### Reporting quality of included studies

**Fig. 3:**
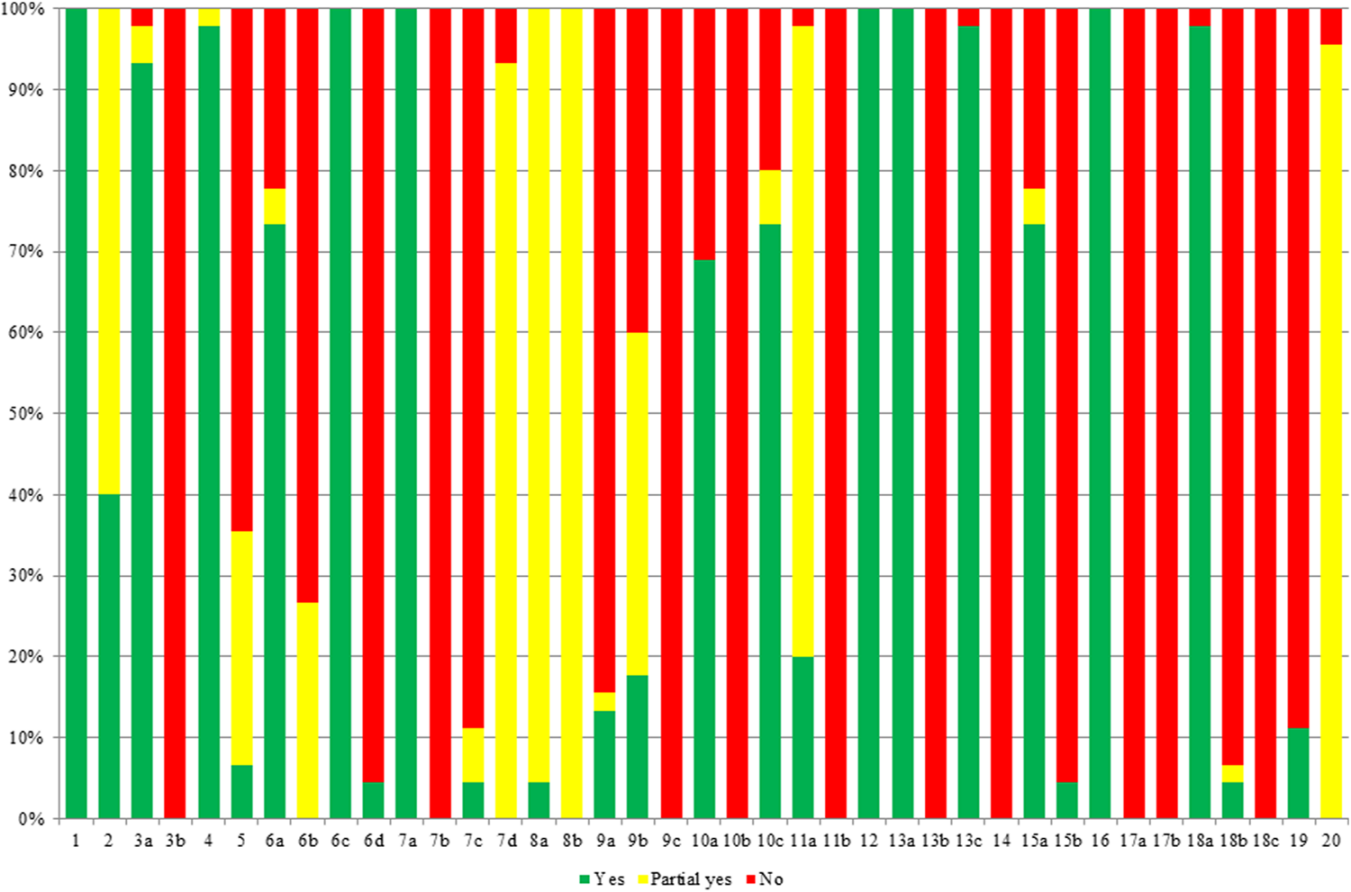
Reporting quality assessment by the ARRIVE guideline

A summary of the ARRIVE guideline results is demonstrated in Table 4 and Fig. 3. No study fulfilled all 39 items of ARRIVE guideline. Merely three studies (7%) described the complete ethical statement. Two studies (4%) drew a time chart or flow chart (Fig. 3(6d)), described the procedure implementation place (Fig. 3(7c)), provided complete details of the animals used (Fig. 3(8a)), explained the reason why some animals or data were not included (Fig. 3(15b)), and commented on the study limitations (Fig. 3(18b)). Six studies (13%) reported housing (Fig. 3(9a)), and eight studies (18%) provided adequate husbandry conditions (Fig. 3(9b)). Nine studies (20%) mentioned randomized grouping. Even worse, there were 10 items even achieving a 100% “no,” including the explanation of how and why the animal species and model were used (Fig. 3(3b)), the description of the procedure implementation time (Fig. 3(7b)), welfare-related evaluations, interventions that were carried out throughout the experiment (Fig. 3(9c)), detailed sample size calculation (Fig. 3(10b)), the order in which the animals in the different experimental groups were treated and assessed (Fig. 3(11b)), the unit of analysis for each dataset specially (Fig. 3(13b)), an offer of the baseline data of experimental animals (Fig. 3(14)), details of important adverse events in each group (Fig. 3(17a)), modifications to the experimental protocols (Fig. 3(17b)), and any implications of your experimental methods or findings for the 3Rs (Fig. 3(18c)), while there were 6 items accurately reported, achieving a 100% “yes,” including a title that accurately described the content of the article (Fig. 3(1)); a study design that mentioned experimental unit (Fig. 3(6c)); an experimental procedure that provided precise details of drug formulation and dose, site and route of administration, anesthesia used, surgical procedure, and method of euthanasia (Fig. 3(7a)); a clear definition that the primary and secondary experimental outcomes were assessed (Fig. 3(12)); details of the statistical methods used for each analysis (Fig. 3(13a)); and the reports on the results of each analysis and accuracy of measures (Fig. 3(16)). Overall, in the 39 items of ARRIVE guideline, 14 (35.90%) items were rated as “yes” in more than 50% of the included studies. The inter-rater reliability was excellent between the two assessors (kappa = 0.95).

**Table 4:** Reporting quality evaluation by the ARRIVE guideline

Reporting item	Number	Recommendation	Yes	Partial yes	No
Title	1	Provide as accurate and concise a description of the content of the article as possible	45 (100%)	0	0
Abstract	2	Provide an accurate summary of the background, research objectives (including details of the species or strain of animal used), key methods, principal findings, and conclusions of the study	18 (40%)	27 (60%)	0
Introduction					
Background	3	a. Include sufficient scientific background (including relevant references to previous work) to understand the motivation and context for the study and explain the experimental approach and rationale	42 (93.33%)	2 (4.44%)	1 (2.22%)
		b. Explain how and why the animal species and model being used can address the scientific objectives and, where appropriate, the study’s relevance to human biology	0	0	45 (100%)
Objectives	4	Clearly describe the primary and any secondary objectives of the study, or specific hypotheses being tested	44 (97.78%)	1 (2.22%)	0
Methods					
Ethical statement	5	Indicate the nature of the ethical review permissions, relevant licences (e.g., Animal [Scientific Procedures] Act 1986), and national or institutional guidelines for the care and use of animals, that cover the research	3 (6.67%)	13 (28.89%)	29 (64.44%)
Study design	6	a. The number of experimental and control groups	33 (73.33%)	2 (4.44%)	10 (22.22%)
		b. Any steps taken to minimize the effects of subjective bias when allocating animals to treatment (e.g., randomization procedure) and when assessing results (e.g., if done, describe who was blinded and when)	0	12 (26.67%)	33 (73.33%)
		c. The experimental unit (e.g., a single animal, group, or cage of animals)	45 (100%)	0	0
		d. A timeline diagram or flow chart can be useful to illustrate how complex study designs were carried out	2 (4.44%)	0	43 (95.56%)
Experimental procedures	7	a. How (e.g., drug formulation and dose, site and route of administration, anesthesia and analgesia used [including monitoring], surgical procedure, method of euthanasia). Provide details of any specialist equipment used, including supplier(s)	45 (100%)	0	0
		b. When (e.g., time of day)	0	0	45 (100%)
		c. Where (e.g., home cage, laboratory, water maze)	2 (4.44%)	3 (6.67%)	40 (88.89%)
		d. Why (e.g., rationale for choice of specific anesthetic, route of administration, drug dose used)	0	42 (93.33%)	3 (6.67%)
Experimental animals	8	a. Provide details of the animals used, including species, strain, sex, developmental stage (e.g., mean or median age plus age range), and weight (e.g., mean or median weight plus weight range)	2 (4.44%)	43 (95.56%)	0
		b. Provide further relevant information such as the source of animals, international strain nomenclature, genetic modification status (e.g., knockout or transgenic), genotype, health/immune status, drug- or test-naive, and previous procedures	0	45 (100%)	0
Housing and husbandry	9	a. Housing (e.g., type of facility, e.g., specific pathogen free (SPF); type of cage or housing; bedding material; number of cage companions; tank shape and material, etc., for fish)	6 (13.33%)	1 (2.22%)	38 (84.44%)
		b. Husbandry conditions (e.g., breeding program; light/dark cycle; temperature; quality of water, etc., for fish; type of food; access to food and water; environmental enrichment)	8 (17.78%)	19 (42.22%)	18 (40%)
		c. Welfare-related assessments and interventions that were carried out before, during, or after the experiment	0	0	45 (100%)
Sample size	10	a. Specify the total number of animals used in each experiment and the number of animals in each experimental group	31 (68.89%)	0	14 (31.11%)
		b. Explain how the number of animals was decided. Provide details of any sample size calculation used	0	0	45 (100%)
		c. Indicate the number of independent replications of each experiment, if relevant	33 (73.33%)	3 (6.67%)	9 (20%)
Allocating animals to experimental groups	11	a. Give full details of how animals were allocated to experimental groups, including randomization or matching if done	9 (20%)	35 (77.78%)	1 (2.22%)
		b. Describe the order in which the animals in the different experimental groups were treated and assessed	0	0	45 (100%)
Experimental outcomes	12	Clearly define the primary and secondary experimental outcomes assessed (e.g., cell death, molecular markers, behavioral changes)	45 (100%)	0	0
Statistical methods	13	a. Provide details of the statistical methods used for each analysis	45 (100%)	0	0
		b. Specify the unit of analysis for each dataset (e.g., single animal, group of animals, single neuron)	0	0	45 (100%)
		c. Describe any methods used to assess whether the data met the assumptions of the statistical approach	44 (97.98%)	0	1 (2.22%)
Results					
Baseline data	14	For each experimental group, report relevant characteristics and health status of animals (e.g., weight, microbiological status, and drug- or test-naive) before treatment or testing (this information can often be tabulated)	0	0	45 (100%)
Numbers analyzed	15	a. Report the number of animals in each group included in each analysis. Report absolute numbers (e.g., 10/20, not 50%)	33 (73.33%)	2 (4.44%)	10 (22.22%)
		b. If any animals or data were not included in the analysis, explain why	2 (4.44%)	0	43 (95.56%)
Outcomes and estimation	16	Report the results for each analysis carried out, with a measure of precision (e.g., standard error or confidence interval)	45 (100%)	0	0
Adverse events	17	a. Give details of all the important adverse events in each experimental group	0	0	45 (100%)
		b. Describe any modifications to the experimental protocols made to reduce adverse events	0	0	45 (100%)
Discussion					
Interpretation/scientific implications	18	a. Interpret the results, taking into account the study objectives and hypotheses, current theory, and other relevant studies in the literature	44 (97.78%)	0	1 (2.22%)
Interpretation/scientific implications Generalizability/ translation	18 19	b. Comment on the study limitations including any potential sources of bias, any limitations of the animal model, and the imprecision associated with the results	2 (4.44%)	1 (2.22%)	42 (93.33%)
		c. Describe any implications of your experimental methods or findings for the replacement, refinement, or reduction (the 3Rs) of the use of animals in research	0	0	45 (100%)
		Comment on whether, and how, the findings of this study are likely to translate to other species or systems, including any relevance to human biology	5 (11.11%)	0	40 (88.89%)
Funding	20	List all funding sources (including grant number) and the role of the funder(s) in the study	0	43 (95.56%)	2 (4.44%)

## Discussion

Experimental researches involving animal models play a crucial role in scientific innovation provided that the experiments are designed, performed, interpreted, and reported well (Bezdjian et al. 2018). Hitherto, an increasing number of experimental researches have reported that BHD is beneficial for CIRI. It has been reported that BHD can protect neurons from ischemic injury, reduce infarction volumes, and stimulate neural proliferation (Zhang et al. 2018). BHD has exhibited the profile as a potential target medicine for the treatment of ischemic stroke or CIRI in facilitating the translation of basic science to the clinical application. However, the conclusions and results may be impeded due to the methodological flaws and poor reporting of experimental researches. Hence, this study aims to assess the methodological and reporting quality of experimental research concerning Buyang Huanwu decoction for mitigating CIRI in rats, to provide useful suggestions for the implementation of the future reviewers and researchers. Unfortunately, the results revealed some limitations in the quality of methodology and reporting, suggesting the need for an improvement in quality in the future.

The methodological quality of included studies is assessed using the SYRCLE tool. The SYRCLE RoB tool (Hooijmans et al. 2014), with 10 items and 22 sub-items, is considered to have high reliability and practicability to evaluate the methodological quality of animal experiments. It not only can assess the risk of bias and improve transparency in the animal research process, but also include a comprehensive user guide. As the sample size of most animal experiments is relatively smaller than that of clinical trials, therefore, necessary baseline characteristics and adequate timing of disease induction for animal experimental disease modeling should be determined to reduce baseline imbalance. In our previous experimental research (Mei et al. 2022, 2020; Yang, et al. 2021), we observed that the 90 min and 24 h may be the appropriate lasting time of MCAO and reperfusion in rat stroke model to mimic the clinical injury of cerebral ischemia and reperfusion. Adequate randomization, allocation concealment, and blinding are suggested to be implemented carefully to reduce the risk of selection bias, performance bias, and detection bias. Also, incomplete outcome data should be reported to reduce the risk of attrition bias, including the appropriate imputations and reasons for missing outcome data. Adherence to a well-developed protocol can reduce the risk of reporting bias. However, these items are not well explained because of a database of registered animal research protocols is not yet publicly accessible. In addition to the above, some other sources of bias need to be paid attention including the contamination, inappropriate influence of funders, unit of analysis errors, design-specific risks of bias, and new animals added to the groups to replace dropouts from the original population.

As for the reporting quality of included studies, we assess it by the ARRIVE guideline. The ARRIVE guideline (Kilkenny et al. 2012), with 20 items and 39 sub-items, aims to fill the gap lacking a set of comprehensive animal research reporting guidelines. It involves important information of animal experiments and promotes substantial improvements in methods used in in vivo animal research. The ARRIVE guideline has been endorsed by over 300 research journals around the world in 2014. Nowadays, the ARRIVE guideline 2.0 has been published in 2020 (Percie du Sert et al. 2020). According to this guideline, it is necessary to explain the reason of using the animal species and model and the study’s relevance to human biology. The full detailed description of the experimental procedures, including the implementation time of procedures, the reason for the route of administration, and the drug dose selection, is the key to ensure accurate experimental reproduction. Baseline data of experimental animals is essential for the results to be comparable. The authors should precisely and explicitly record the details of the animals used including source, species, strain, international strain nomenclature, sex, developmental stage, weight, genetic modification status, genotype, health/immune status, and previous procedures. The information of relevant characteristics and health status of animals before treatment or testing can often be tabulated. The risk of overestimating intervention benefits may be induced by inadequate samples. An adequate sample size with enough statistical power can easily detect statistical differences between groups. So, it is necessary to calculate the sample size before the experiment. It is also significant to focus on adverse events in animal researches to determine the pros and cons of an intervention. In addition, the significance of the study is closely relevant to the utilization rate and conversion of the study. It is also necessary to discuss whether and how these study findings can translate into other species or systems.

The results suggested that methodological and reporting quality should be controlled strictly during the design, implementation, interpretation, and report of experimental research. The SYRCLE tool and ARRIVE guideline could be used to assess the whole process of the animal experiment both rigorously and comprehensively. Nowadays, most studies have low methodological and reporting quality. A recent research (Zhang et al. 2019a) shows that Chinese basic medical researchers have a low awareness and use rates of the SYRCLE tool and the ARRIVE guideline, leading to the low quality of animal studies in Chinese journals (Wang et al. 2019). In this review, 80% of studies are published in Chinese-language journals. Therefore, it is necessary to take specific measures to promote and popularize these standards and specifications and to introduce them into guidelines of Chinese domestic journals as soon as possible so as to raise awareness and increase utilization rates of researchers and journal editors.

Although we follow strict procedures in this review, it still has some limitations. Firstly, to some extent, the quality defects of the included studies affect our evaluation results. Secondly, the literatures included in our study are only in Chinese and English languages, which may have a linguistic bias. Thirdly, this is the first time that the evaluation analysts use the SYRCLE tool and ARRIVE guideline. The evaluation of many items involved is inevitably subjective and may have led to bias. Finally, the findings may not be applicable to other traditional Chinese medicine trials due to the interventions merely contained BHD and the species restricted to rats.

## Conclusions

BHD has been used increasingly in the preclinical researches to treat CIRI, which seems to be a potential treatment option for alleviating CIRI in patients. However, based on the SYRCLE tool and ARRIVE guideline, the methodological and reporting quality of BHD against CIRI were poor. Our findings will urge journal editors; the researchers, clinicians, and reviewers; or funding agencies to pay more attention to address these deficiencies and strengthen training to meet relevant requirements on methodologies and reporting quality by strictly adopting and adhering to well-developed reporting guidelines: the SYRCLE tool and ARRIVE guideline.

## Data Availability

All data are available from the first author (Xiangyu Chen) on reasonable request.

## Abbreviations

5-HT: 5-Hydroxytryptamine
6-keto-PGF1α: 6-Keto prostaglandin F1α
AMPK: Adenosine monophosphate–activated protein kinase
APTT: Activated partial thromboplastin time
Arg-1: Arginase-1
ARRIVE: Animal Research: Reporting In Vivo Experiments
ASC: Apoptosis-associated speck-like protein
ATG12: Autophagy-related gene 12
Bax: Bcl2-associated X
BBB: Blood-brain barrier
Bcl-2: B-cell lymphoma 2
BDNF: Brain-derived neurotrophic factor
BHD: Buyang Huanwu decoction
BrdU: 5-Bromo-2′-deoxyuridine
BWT: Beam walking test
Caspases: Cysteine aspartase
CBF: Cerebral blood flow
CBM: China Biology Medicine Database
CD206: Cluster of differentiation 206
CD86: Cluster of differentiation 86
CIRI: Cerebral ischemia–reperfusion injury
CNKI: China National Knowledge Infrastructure
Cx43: Connexin 43
cyt-c: Cytochrome C
DCX: Doublecortin on the X chromosome
Drp1: Dynamin-related protein 1
EEG: Electroencephalograph
elF2α: Eukaryotic initiation factor 2α
Embase: Excerpta Medica Database
ENaC: Epithelial sodium channel
FDA: Food and Drug Administration
FIB: Fibrinogen
Fis1: Fission 1
FPR2: Formyl peptide receptor 2
GAP-43: Growth-associated protein 43
GFAP: Glial fibrillary acidic protein
GPX/GSH-Px: Glutathione peroxidase
GRP78: Glucose-regulated protein 78
GSH: Glutathione
GSK: Glycogen synthase kinase
GST: Glutathione *S*-transferase
HIF: Hypoxia-inducible factor
IFN: Interferon
IL: Interleukin
iNOS: Inducible nitric oxide synthase
ITGαvβ3: Integrin αvβ3
IκBα: Inhibitor α of NF-κB
LC3: Microtubule-associated protein light chain 3
LDH: Lactate dehydrogenase
MDA: Malondialdehyde
MMP: Matrix metalloproteinase
mRNA: Messenger ribonucleic acid
mTOR: Mammalian target of rapamycin
MVD: Microvascular density
MWM: Morris water maze
NeuN: Neuronal nuclei
NF-κB: Nuclear factor-kappa B
NO: Nitric oxide
NOX2: Nicotinamide adenine dinucleotide phosphate II (NADPH) oxidase 2
PAF: Platelet activating factor
p-AKT: Phosphothreonine kinase
PDK1: Phosphoinositide-dependent protein kinase 1
PERK: Protein kinase RNA-like endoplasmic reticulum kinase
PI3K: Phosphatidylinositol 3-kinase
PT: Prothrombin time
PTEN: Phosphatase and tensin homolog
ROS: Reactive oxygen species
RvD1: Resolvin D1
SAB: Spontaneous alternation behavior
SD: Sprague Dawley
SIRT1: Sirtuin-1
SOD: Superoxide dismutase
SYN: Synaptophysin
SYRCLE: Systematic Review Centre for Laboratory Animal Experimentation
TGF-β: Transforming growth factor-β
TLR4: Toll-like receptor 4
TNF: Tumor necrosis factor
TT: Thrombin time
TUNEL: Terminal-deoxynucleotidyl transferase–mediated nick end labeling
TXB2: Thromboxane B2
ULK1: UNC-51-like kinase 1
VEGF: Vascular endothelial growth factor
VIP: VIP Database for Chinese Technical Periodicals
WBV: Whole blood viscosity
Wnt3a: Wingless-type MMTV integration site family member 3a
ZO-1: Zonula occluden-1
